# Aryl Hydrocarbon Receptor-Mediated Perturbations in Gene Expression during Early Stages of CD4^+^ T-cell Differentiation

**DOI:** 10.3389/fimmu.2012.00223

**Published:** 2012-08-06

**Authors:** Diana Rohlman, Duy Pham, Zhen Yu, Linda B. Steppan, Nancy I. Kerkvliet

**Affiliations:** ^1^Kerkvliet Laboratory, Environmental and Molecular Toxicology, Oregon State UniversityCorvallis, OR, USA; ^2^Environmental Health Sciences Center, Oregon State UniversityCorvallis, OR, USA

**Keywords:** aryl hydrocarbon receptor, 2,3,7,8-tetrachlorodibenzo-*p*-dioxin, CD4^+^ T-cell differentiation, gene expression, IL-22

## Abstract

Activation of the aryl hydrocarbon receptor (AhR) by its prototypic ligand, 2,3,7,8-tetrachlorodibenzo-*p*-dioxin (TCDD), mediates potent suppression of T-cell dependent immune responses. The suppressive effects of TCDD occur early during CD4^+^ T-cell differentiation in the absence of effects on proliferation and have recently been associated with the induction of AhR-dependent regulatory T-cells (Treg). Since AhR functions as a ligand-activated transcription factor, changes in gene expression induced by TCDD during the early stages of CD4^+^ T-cell differentiation are likely to reflect fundamental mechanisms of AhR action. A custom panel of genes associated with T-cell differentiation was used to query changes in gene expression induced by exposure to 1 nM TCDD. CD4^+^ T-cells from AhR^+/+^ and AhR^−/−^ mice were cultured with cytokines known to polarize the differentiation of T-cells to various effector lineages. Treatment with TCDD induced the expression of *Cyp1a1*, *Cyp1b1*, and *Ahrr* in CD4^+^ T-cells from AhR^+/+^ mice under all culture conditions, validating the presence and activation of AhR in these cells. The highest levels of AhR activation occurred under Th17 conditions at 24 h and Tr1 conditions at 48 h. Unexpectedly, expression levels of most genes associated with early T-cell differentiation were unaltered by AhR activation, including lineage-specific genes that drive CD4^+^ T-cell polarization. The major exception was AhR-dependent up-regulation of *Il22* that was seen under all culture conditions. Independent of TCDD, AhR down-regulated the expression of *Il17a* and *Rorc* based on increased expression of these genes in AhR-deficient cells across culture conditions. These findings are consistent with a role for AhR in down-regulation of inflammatory immune responses and implicate IL-22 as a potential contributor to the immunosuppressive effects of TCDD.

## Introduction

Activation of the transcription factor, aryl hydrocarbon receptor (AhR), by the prototypic ligand 2,3,7,8-tetrachlorodibenzo-*p*-dioxin (TCDD) has been shown to exert potent immunosuppressive effects in a variety of T-cell dependent disease models. TCDD suppresses Th1-dependent autoimmune diseases such as Type 1 diabetes in the non-obese diabetic (NOD) mouse (Kerkvliet et al., 2009) and experimental autoimmune uveoretinitis (Zhang et al., 2010). TCDD also suppresses the Th17-mediated response in both experimental autoimmune encephalomyelitis (EAE) and colitis (Quintana et al., 2008; Benson and Shepherd, 2011). These potent immunosuppressive effects are also conserved in non-autoimmune disease models. The Th1-driven cytotoxic T-lymphocyte (CTL) and alloantibody responses to allogeneic P815 tumor cells are dose-dependently suppressed by TCDD treatment (Kerkvliet et al., 1996), as are CTL and antibody responses to influenza virus infection (Warren et al., 2000). Treatment with TCDD also suppresses Th2-dependent cytokine and OVA-specific antibody production (Shepherd et al., 2000; Nohara et al., 2002), as well as several Th2-dependent allergic diseases including atopic dermatitis (Fujimaki et al., 2002), dust mite-induced asthma (Luebke et al., 2001), and peanut allergy (Schulz et al., 2011).

One of the mechanisms by which TCDD suppresses adaptive immunity occurs via activation of AhR in differentiating CD4^+^ T-cells. This was demonstrated using an acute parent-into-F1 graft-versus-host (GVH) model wherein the suppression of donor CTL development was contingent upon the donor CD4^+^ T-cells expressing AhR (Kerkvliet et al., 2002). Furthermore, optimal suppression of the CTL response occurred when TCDD was administered within 3 days of alloantigen stimulation – the time during which donor CD4^+^ T-cells undergo differentiation (Kerkvliet et al., 1996). Phenotypic and functional analysis of the differentiating allospecific donor cells suggested that AhR activation was driving the development of an immunosuppressive regulatory T-cell (Treg), characterized by a CD4^+^CD25^high^Foxp3^neg^CTLA-4^+^CD62L^low^IL-10^+^ phenotype (Funatake et al., 2005; Marshall et al., 2008). Subsequently, TCDD has also been shown to induce Foxp3^+^ Tregs and Tr1 cells *in vitro* (Apetoh et al., 2010; Gandhi et al., 2010) and to increase the frequency of Foxp3^+^ CD4^+^ T-cells *in vivo* in several models of immune-mediated disease (Quintana et al., 2008; Kerkvliet et al., 2009; Takamura et al., 2010; Zhang et al., 2010; Benson and Shepherd, 2011; Schulz et al., 2011; Singh et al., 2011).

Based on its role as a transcription factor, activation of AhR in CD4^+^ T-cells may directly alter CD4^+^ T-cell differentiation by influencing gene expression during early differentiation events. The likelihood of such effects is high given the large number of immune-related genes that contain dioxin response elements (DRE; Sun et al., 2004; Frericks et al., 2008; Kerkvliet, 2009). In the present studies, we characterized the influence of TCDD-activated AhR on gene expression during CD4^+^ T-cell differentiation under Th0, Th1, Treg, Tr1, and Th17 polarizing conditions. We utilized a custom panel of 48 genes that have been associated with AhR activation, T-cell differentiation, and/or Treg induction (Table 1). CD4^+^ T-cells were obtained from AhR^+/+^ and AhR-deficient (AhR^−/−^) mice, allowing us to validate the AhR-dependence of TCDD’s effects. In addition, differences in gene expression between vehicle-treated cultures of AhR^+/+^ and AhR^−/−^ CD4^+^ T-cells identified genes that are regulated by AhR during T-cell activation in the absence of an exogenous ligand.

**Table 1:** **Panel of genes used to evaluate AhR regulation of gene expression in CD4^+^ T-cells**.

Gene symbol	Gene name	RefSeq
*A2br*	Adora2b	NM_007413
*Actb*	Actb	NM_007393.3
*Ahr*	AhR	NM_013464.4
*Ahrr*	AhRR	NM_009644
*Bach2*	Bach2	NM_007521
*Cd27*	CD27	NM_001033126.2
*Cd40lg*	Tnfsf5	NM_011616.2
*Cd69*	CD69	NM_001033122
*Ctla4*	CTLA-4	NM_009843.3
*Cyp1a1*	Cyp1a1	NM_009992.2
*Cyp1b1*	Cyb1b1	NM_009994.1
*Entpd1*	Entpd1/CD39	NM_009848
*Fasl*	Tnfsf6	NM_010177.3
*Foxp3*	Foxp3	NM_054039.1
*Gata3*	Gata3	NM_008091.3
*Gzmb*	Gzmb	NM_013542
*Hmox1*	Hmox1	NM_010442.1
*Icos*	ICOS	NM_017480.1
*Ifng*	IFNγ	NM_008337.1
*Il2*	IL-2	NM_008366.2
*Il4*	IL-4	NM_021283.1
*Il6*	IL-6	NM_031168.1
*Il10*	IL-10	NM_010548.1
*Il10ra*	IL-10Ra	NM_008348.2
*Il12a*	IL-12a	NM_008351.1
*Il12b*	IL-12b	NM_008352.2
*Il12rb2*	IL-12Rβ2	NM_008354.1
*Il17a*	IL-17A	NM_010552
*Il-21*	IL-21	NM_021782.2
*Il-21r*	IL-21R	NM_021887.1
*Il22*	IL-22	NM_016971.1
*Il27ra*	IL-27Rα	NM_016671.2
*Il2ra*	IL-2Rα/CD25	NM_008367.2
*Maf*	c-MAF	NM_001025577.2
*Nfatc2*	Nfatc2	NM_010899.2
*Nfe2l2*	Nfe212	NM_010902
*Pdcd1*	PD-1	NM_008798.2
*Prdm1*	Prdm1	NM_007548
*Rorc*	RORγ	NM_011281.1
*Socs3*	Socs3	NM_007707.2
*Stat4*	Stat4	NM_011487
*Tbx21*	Tbx21/Tbet	NM_019507.1
*Tgfb1*	TGFβ1	NM_011577.1
*Tgfb3*	TGFβ3	NM_009368.1
*Tgfbr1*	TGFβR1	NM_009370.2
*Tgfbr2*	TGFβR2	NM_009371.2
*Tnf*	TNFα	NM_013693.1
*Tnfrsf4*	Tnfrsf4/OX40	NM_011659

*Genes were chosen based on association with T-cell activation and differentiation, or genes that have been previously identified to be responsive to TCDD *in vivo**.

## Materials and Methods

### Animals

B6.PL-Thy1a/CyJ mice (Thy1.1^+/+^, AhR^+/+^) and B6.129-AhR^tm1Bra^/J (Thy1.1^+/+^, AhR^−/−^) mice were bred and maintained under specific pathogen-free conditions at the Laboratory Animal Resource Center at Oregon State University (Corvallis, OR, USA). All animal procedures were approved by the Institutional Animal Care and Use Committee.

### CD4^+^ T-cell cultures

Spleens were aseptically removed and processed into single-cell suspensions via dissociation between the frosted ends of microscope slides. Red blood cells and dead cells were removed by hypotonic water lysis. CD4^+^ T-cells were isolated by negative selection using a CD4^+^ T-cell isolation kit and an autoMACS separator (Miltenyi Biotec; Auburn, CA, USA). T-cells were cultured in RPMI 1640 media (Invitrogen; Carlsbad, CA, USA), supplemented with 10% fetal bovine serum (HyClone; Logan, UT, USA), 10 mM HEPES (Invitrogen), 50 μg/ml gentamicin (Invitrogen), and 50 μM 2-β-mercaptoethanol (Sigma; St. Louis, MO, USA). At the time of culture initiation, cells were treated with 1 nM TCDD (dissolved in DMSO) or 0.001% DMSO (vehicle). The 1 nM concentration of TCDD used in these studies was sufficient to induce maximum activation of AhR in T-cells as reflected in expression of known AhR-regulated genes *Cyp1a1*, *Cyp1b1*, and *Ahrr* (unpublished data).

CD4^+^ T-cells (1 × 10^6^ cells/well) were activated with soluble anti-CD3 (0.5 μg/ml) and anti-CD28 (2.5 μg/ml) or plate-bound anti-CD3 (2 μg/ml) and anti-CD28 (2 μg/ml) in a 24-well plate. For Th1 conditions, anti-IL-4 (10 μg/ml) and IL-12 (3 ng/ml) was added to each well. For Treg polarizing conditions, TGFβ1 (3 ng/ml) was added. In addition to TGFβ1, IL-27 (25 ng/ml), or IL-6 (15 ng/ml) was added for Tr1 or Th17 polarizing conditions, respectively. All reagents for T-cell polarization were purchased from eBioscience. T-cells cultured under Th0 conditions received no exogenous cytokines.

For some genes (*Il22*, *Il17a*, *Rorc*), protein levels were also measured. Whole splenocytes or purified CFSE-labeled CD4^+^ T-cells were cultured under Th17 conditions. After 48 h, cells were treated for 6 h with a Cell Stimulation Cocktail (eBioscience) containing phorbol 12-myristate 13-acetate, ionomycin, Brefeldin A, and monensin. The cells were then stained for intracellular protein prior to flow cytometric analysis as described below. Supernatants were collected for ELISA analysis.

### RNA isolation and qPCR

CD4^+^ T-cells were harvested at 24 and 48 h and flash frozen in liquid nitrogen. Cell pellets were stored at −80°C. RNA was isolated using the RNeasy Mini Kit #74104 (Qiagen; Valencia, CA, USA), with on-column DNase digestion (Qiagen #79254) performed twice to ensure complete elimination of genomic DNA. RNA concentrations and purity were determined using the NanoDrop ND-1000 UV-Vis Spectrophotometer (ThermoScientific). The Reaction Ready First Strand cDNA synthesis kit #C-01/C-03 (SA Biosciences, formerly SuperArray; Frederick, MD, USA) was used to synthesize cDNA in a two-step PCR reaction. A minimum of 200 ng RNA was used for all reactions. Following reverse transcription, samples were stored at −80°C.

An ABI PRISM 7500 Real-Time PCR system (Applied Biosystems) was used for all qPCR reactions. SYBR Green/ROX qPCR Master Mix (SA Bioscience) was used to prepare a 25 μl reaction mix consisting of 12.5 μl master mix, cDNA equivalent to 2 ng RNA, 1 μl primer, and RNase-free H_2_0 to obtain the final volume. All primers were obtained from SA Biosciences. Sequence accession numbers are shown in Table 1. The qPCR settings were as follows: 10 min at 95°C, followed by 40 cycles of 15 s at 95°C, followed by 1 min at 60°C. This was followed by a standard dissociation step of 15 s at 95°C to generate a melting curve. A no-reverse transcription (NRT) control was included to validate the absence of genomic DNA from all samples.

### qPCR data analysis

Data were analyzed using ABI 7500 analysis software. Genes that were not detected within 40 cycles received a value of 40. Cycle numbers were then normalized to *Actb* to calculate ΔCt. The data were analyzed as either 1/ΔCt or 1/ΔCt × 100; all other data were presented as fold change. Fold changes were calculated by the following formulas:

$$\begin{array}{rcll} & \text{Δ}Ct_{\text{experimental}}-\text{Δ}Ct_{\text{control}}=\text{ΔΔ}Ct & & \\ & 2^{\left(-\text{ΔΔ}Ct\right)}=\text{fold}\;\text{change} & & \end{array}$$

When analyzing gene expression across polarizing conditions, fold changes were calculated relative to Th0 as follows:

$$\begin{array}{rcll} & \text{Δ}Ct_{\left(Th1;Th17;Treg;Trl\right)}-\text{Δ}Ct_{Th0}=\text{ΔΔ}Ct & & \\ & 2^{\left(-\text{ΔΔ}Ct\right)}=\text{fold change} & & \end{array}$$

### Flow cytometric analysis

The following antibodies were purchased from eBioscience: CD4-PE (GK1.5), IL-22-PE (1H8PWSR), IL-17A-APC (eBio17B7), and RORγ-PE (AFKJS). CD4-APC (GK1.5) and carboxyfluorescein succinimidyl ester (CFSE) were purchased from Invitrogen. Splenocytes or purified CFSE-labeled CD4^+^ T-cells were resuspended in PAB (PBS, 1% BSA, 0.1% sodium azide), and incubated with PE- or APC-labeled anti-CD4 antibody for 10 min. After two washes, cells were resuspended in Fixable Viability Dye efluor 780 (eBioscience) for 30 min. The BD Cytofix/Cytoperm buffer set or the Foxp3 Staining Buffer set (eBioscience) was used for intracellular staining. Control samples that contained all of the antibodies except the one of interest (fluorescence minus one) were used as negative staining controls. A minimum of 150,000 viable CD4^+^ T-cells were collected per sample on a Beckman Coulter FC-500 flow cytometer. Compensation and analysis of data were performed in WinList (Verity Software, Version 6.0).

### ELISA

Supernatants from purified CD4^+^ T-cells and splenocyte cultures were collected after 48 h. IL-22 production was quantified using the IL-22 Ready-Set-Go^®^ ELISA kit from eBioscience, according to manufacturer’s instructions.

### Statistical analysis

All treatment groups consisted of a minimum of three biological replicates (two mice per replicate), except where indicated. Each biological sample was treated with vehicle or TCDD in separate wells. Differences due to TCDD treatment were determined using a paired Student’s *t*-test. For comparisons between AhR^+/+^ and AhR^−/−^ CD4^+^ T-cells, or between culture conditions, a non-paired Student’s *t*-test was used. Values were considered statistically significant at *p* ≤ 0.05, in conjunction with a fold change of ≥2 between treatment groups. For *Il22* analysis across treatments, a linear mixed model was utilized using the Mixed procedure of SAS (v. 9.2).

## Results

### Aryl hydrocarbon receptor is present and transcriptionally active during early differentiation of CD4^+^ T-cells

**Figure 1:**
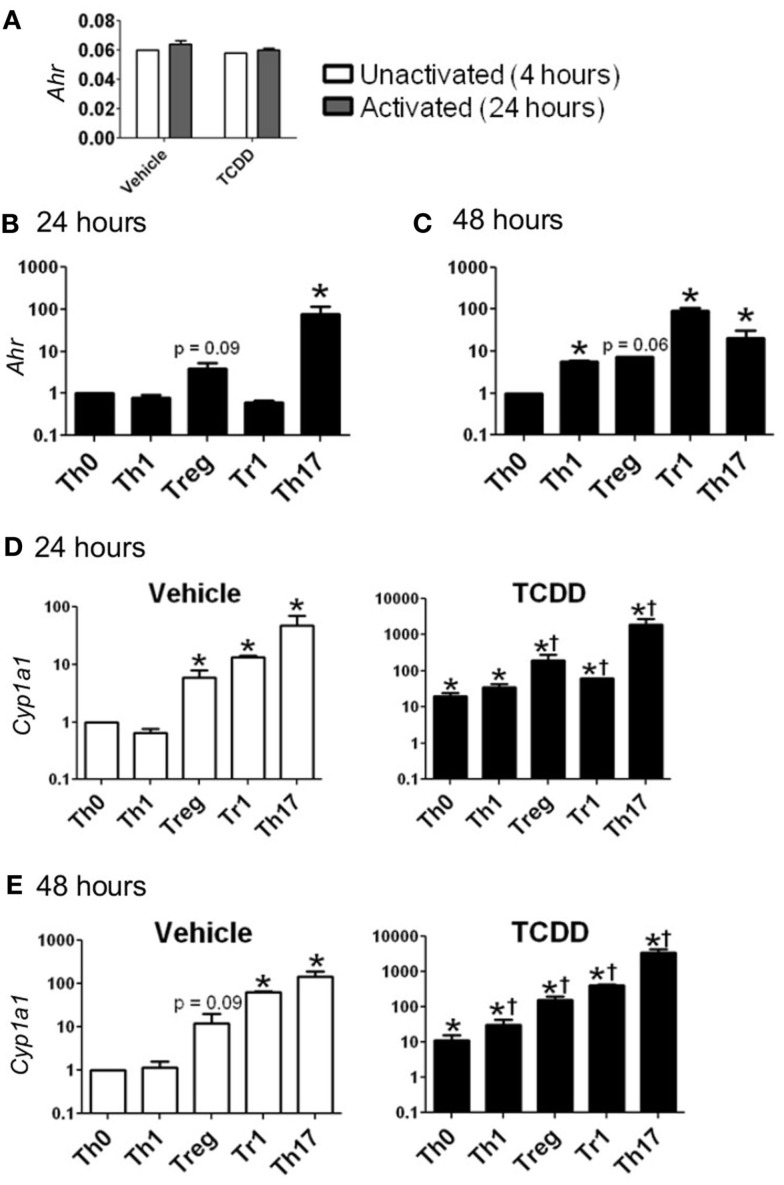
**Expression of *Ahr* and *Cyp1a1* in CD4^+^ T-cells cultured under various conditions**. **(A)** Basal expression of *Ahr* (1/ΔCt) in CD4^+^ T-cells cultured for 4 h without stimulation or 24 h following stimulation with anti-CD3/anti-CD28. **(B,C)** *Ahr* expression in CD4^+^ T-cells cultured for 24 or 48 h with polarizing cytokines as described in Section “Materials and Methods.” Data are presented as fold change relative to Th0 vehicle conditions at 24 or 48 h. **(D,E)** Expression of *Cyp1a1* in vehicle- and TCDD-treated CD4^+^ T-cells cultured for 24 or 48 h under different polarizing conditions. Data are presented as fold change relative to the vehicle-treated Th0 condition at 24 or 48 h. *p ≤ 0.05 relative to either vehicle or TCDD Th0 control within that time point. †*p* ≤ 0.05 relative to the TCDD-treated Th0 condition at 24 or 48 h. Error bars indicate the mean ± SEM; *n* = 3−4 biological replicates.

*Ahr* message was detected in CD4^+^ T-cells cultured under all conditions. Cells activated for 24 h in the absence of exogenous cytokines (Th0 conditions) expressed *Ahr* at a level comparable to that of non-activated cells, indicating that expression of *Ahr* is not up-regulated due to T-cell receptor activation alone (Figure 1A). Likewise, neither Th1 nor Tr1 polarizing conditions produced a significant increase in *Ahr* expression. In contrast, Treg conditions induced a fourfold increase and Th17 conditions induced a 78-fold increase in *Ahr* expression at 24 h (Figure 1B).

At 48 h, *Ahr* expression under Th0 conditions was equivalent to the level of expression seen at 24 h, while expression was up-regulated by 5.6-fold under Th1 conditions and 7.4-fold under Treg polarizing conditions (Figure 1C). Expression of *Ahr* declined under Th17 conditions but remained significantly up-regulated (21-fold) compared to Th0 conditions. *Ahr* expression was highest at 48 h under Tr1 conditions with a fold change of 92 relative to Th0 conditions.

**Figure A1:**
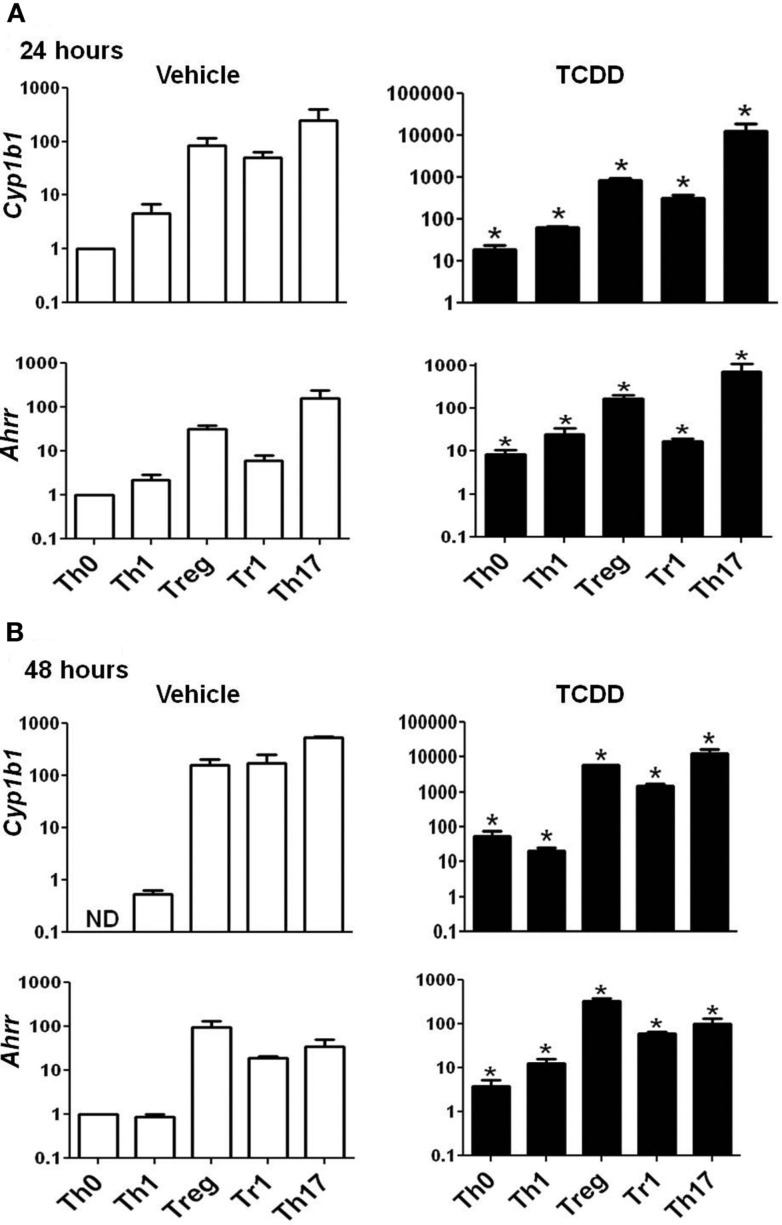
**Expression of *Cyp1b1* and *Ahrr* in AhR^+/+^ CD4^+^ T-cells treated with vehicle or TCDD**. Data are presented as fold change calculated relative to Th0 vehicle at **(A)** 24 or **(B)** 48 h. Expression of *Cyp1b1* at 48 h in Th0 vehicle was greater than 40 cycles; a value of 40 was used to calculate fold changes. Error bars represent the mean ± SEM; *n* = 3−4 biological replicates. *p ≤ 0.05 relative to the vehicle control. ND = not detected.

To determine if the level of *Ahr* message present under different culture conditions was indicative of AhR protein, the ability of TCDD to increase expression of known AhR-regulated genes was examined. Addition of TCDD *per se* did not influence *Ahr* expression except for a small increase that was noted under Treg conditions at 24 h (Table 2). In contrast, addition of TCDD significantly up-regulated *Cyp1a1* expression under all conditions tested (Figures 1D,E), with good correlation between the levels of expression of *Cyp1a1* and *Ahr* at both 24 and 48 h (Figure 1). A similar correlation was observed between expression levels of *Ahr* and expression levels of the AhR-regulated genes *Cyp1b1* and *Ahrr* (Figure A1 in Appendix). These results indicate that sufficient AhR is expressed in CD4^+^ T-cells cultured under any condition to be able to respond to 1 nM TCDD and alter expression of AhR-regulated genes.

**Table 2:** **Influence of TCDD on *Ahr* expression in CD4^+^ T-cells cultured under different polarizing conditions**.

	24 h			48 h		
	Average ΔCt^‡^		Fold change	Average ΔCt		Fold change
	VEH	TCDD	TCDD/VEH	VEH	TCDD	TCDD/VEH
Th0	11.2	12.4	−2.3^†^	12.2	13.4	−2.4^†^
Th1	12.0	11.2	1.7	10.7	11.1	−1.3
Treg	9.8	10.8	−2.0*	8.0	9.4	−2.8^†^
Tr1	12.3	12.8	−1.5	6.7	6.9	−1.2
Th17	4.9	5.3	−1.4	9.0	9.9	−2.0

*^‡^Average cycle number of 3−4 biological replicates, corrected to β-actin*.

^†^NSD; *p ≤ 0.05.

**Figure 2:**
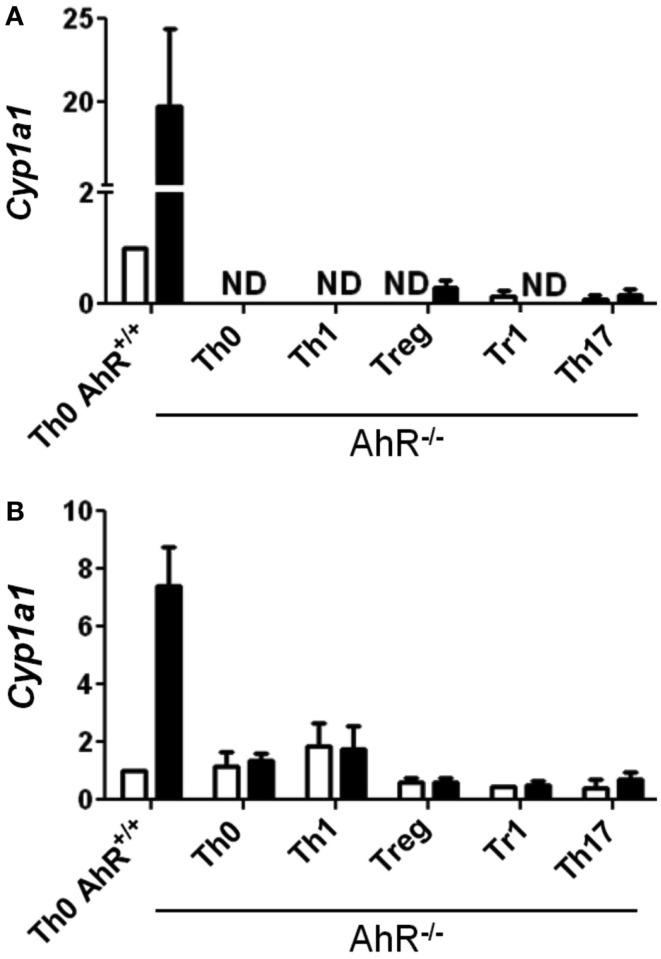
**Expression of *Cyp1a1* in AhR^−/−^ CD4^+^ T-cells**. Data are presented as fold change relative to AhR^+/+^ CD4^+^ T-cells cultured under vehicle-treated Th0 conditions for **(A)** 24 or **(B)** 48 h. Error bars indicate the mean ± SEM; *n* = 3−4 biological replicates, except for 24 h AhR^−/−^ Treg and Th17 conditions, and 48 h AhR^−/−^ Treg and Tr1 conditions, where *n* = 2. *p ≤ 0.05; ND = not detected within 40 cycles.

While TCDD-induced relatively high levels of *Cyp1a1*, low levels of *Cyp1a1* were also seen in the absence of an exogenous ligand in CD4^+^ T-cells cultured under Treg, Tr1, and Th17 polarizing conditions at both 24 and 48 h (Figures 1D,E). These results suggest that the cytokine milieu either contains or is capable of inducing the formation of an AhR ligand in the responding T-cells. Interestingly very low but detectable *Cyp1a1* expression was also seen in AhR-deficient cells especially at 48 h under all polarizing conditions (Figure 2). Addition of TCDD did not up-regulate this expression, validating the absence of functional AhR protein in AhR^−/−^ cells (Figure 2), but implicating an AhR-independent pathway for *Cyp1a1* expression.

### Effect of AhR activation by TCDD on expression of genes involved in CD4^+^ T-cell differentiation

**Figure 3:**
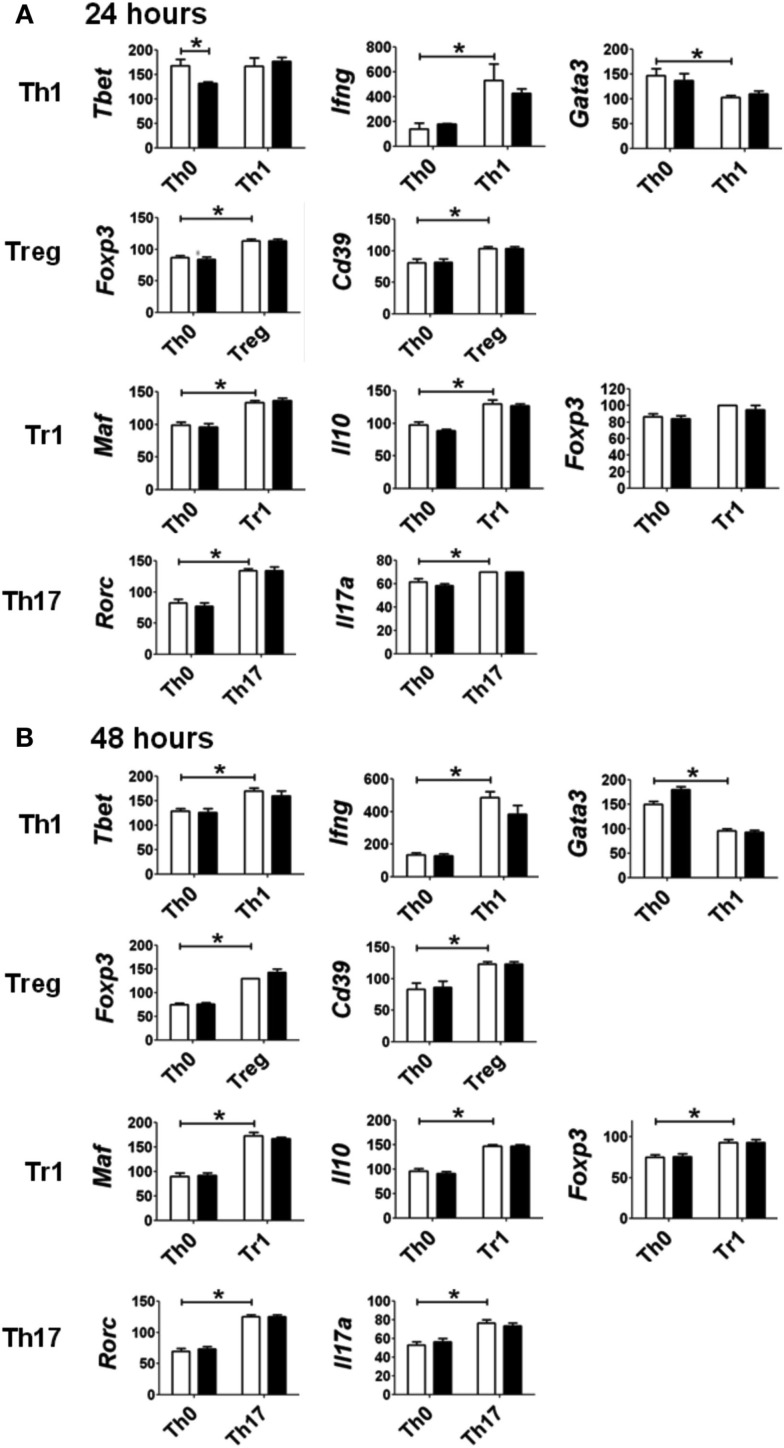
**Expression of genes associated with specific polarization in CD4^+^ T-cells treated with vehicle (white bar) or TCDD (black bar) for (A) 24 or (B) 48 h**. Data are presented as 1/ΔCt × 100. Gene expression under Th0 conditions (no polarizing cytokines) is presented for comparison with gene expression under specific polarizing conditions as described in Section “Materials and Methods.” Error bars represent the mean ± SEM; *n* = 3−4 biological replicates. *p ≤ 0.05.

The pattern of gene expression observed under the various polarizing conditions was consistent with early differentiation events specific to different CD4^+^ T-cell subsets (Figure 3; Zhou et al., 2009; Hermann-Kleiter and Baier, 2010). Under Th1 polarizing conditions, the transcription factor *Tbet* was up-regulated at 48 h, and the associated cytokine *Ifng* was up-regulated at 24 and 48 h when compared to expression levels in non-polarized (Th0) cells. The up-regulation of these Th1-associated genes was concurrent with the down-regulation of *Gata3*, also consistent with Th1 differentiation. Similarly, the transcription factor for T-regulatory cells, *Foxp3*, and the associated Treg marker *Cd39* were up-regulated under Treg polarizing conditions, while Tr1 polarizing conditions induced the expression of *Maf* and *Il10*. *Foxp3* expression was also up-regulated under Tr1 polarizing conditions at 48 h, albeit to a lesser extent than that seen under Treg polarizing conditions. As expected Th17 polarizing conditions produced increased expression of *Rorc* and *Il17* at 24 and 48 h.

**Figure 4:**
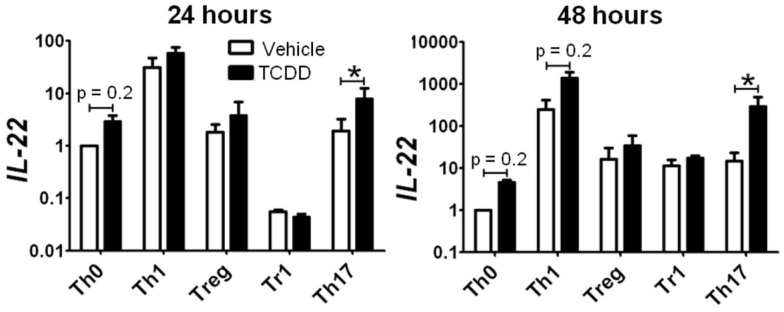
**Influence of TCDD on *Il22* expression under various polarizing conditions**. Data are presented as fold change relative to expression in Th0 vehicle controls at 24 or 48 h. Error bars represent the mean ± SEM; *n* = 3−4 biological replicates. *p ≤ 0.05.

Surprisingly, activation of AhR by TCDD did not significantly alter expression of any of the master regulator genes associated with CD4^+^ T-cell polarization or other genes associated with polarization (Figure 3). In fact, expression of only one gene in the panel of genes tested, *Il22*, was clearly altered by treatment with TCDD, and this effect was seen under all conditions except Tr1 (Table 3). When analyzed over all conditions except Tr1, expression of *Il22* was significantly increased in the presence of TCDD at both 24 (*p* = 0.03) and 48 h (*p* < 0.001). The level of *Il22* expression was highest under Th1 conditions in vehicle-treated cells, and addition of TCDD further increased expression (Figure 4), while the greatest induction of *Il22* expression by TCDD (17.6-fold) occurred under Th17 conditions at 48 h (Table 3). The minimal expression of *Il22* under Tr1 conditions was surprising given the high level of *Ahr* expressed in these cells at 48 h and suggested that the presence of IL-27 as a polarizing cytokine inhibits *Il22* transcription. In fact, Rutz et al. (2011) recently showed that the induction of c-Maf by IL-27 results in downstream repression of *Il22*. Analysis of AhR^−/−^ CD4^+^ T-cells showed no significant increase in *Il22* expression upon treatment with TCDD at either 24 or 48 h under any polarizing condition, verifying that the up-regulation of *Il22* by TCDD was dependent on AhR activation (Table 3).

**Table 3:** **Influence of TCDD on *Il22* expression in AhR^+/^^+^ and AhR^−/^^−^ CD4^+^ T-cells activated under different polarizing conditions**.

Polarizing condition	AhR^+/+^ TCDD/VEH		AhR^−/−^ TCDD/VEH	
	24 h	48 h	24 h	48 h
Th0	2.5	3.8	1.3	−1.8
Th1	2.3	13.7	−1.4	3.1
Treg	2.2	6.4	1.5	1.0
Tr1	−1.1	1.7	−1.9	1.4
Th17	4.6	17.6	−1.1	−1.2
TCDD effect (*p*-value) excluding Tr1 conditions	0.03	<0.001	0.88	0.82

*Data are presented as fold change in TCDD-treated cells relative to vehicle-treated controls at 24 or 48 h. Data represent 3−4 biological replicates, except for AhR^−/−^ cells at 24-h Treg and Th17 and 48-h Treg conditions where *n* = 2. Data were analyzed for significant TCDD-dependent effect across culture conditions, excluding Tr1*.

**Figure 5:**
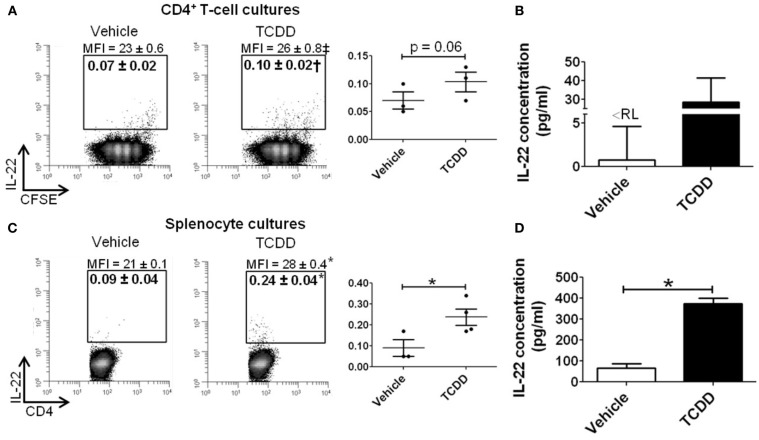
**Influence of TCDD on IL-22 protein in CD4^+^ T-cells cultured under Th17 polarizing conditions for 48 h**. Data shown are gated on viable CD4^+^ T-cells. **(A)** CD4^+^ T-cells were purified and labeled with CFSE prior to activation. Percent CD4^+^IL-22^+^ T-cells are shown. MFI, median fluorescence intensity. Individual biological replicates are represented graphically. Histograms shown are representative of 3−4 biological replicates. **(B)** Concentration of IL-22 in supernatant from purified CD4^+^ T-cell cultures.<RL = below reporting limit. **(C)** Whole spleen cell suspensions were activated and cultured under Th17 polarizing conditions, as in **(A)**. **(D)** Concentration of IL-22 in supernatant from whole splenocyte cultures. Error bars represent the mean ± SEM; *n* = 3−4 biological replicates. †*p* = 0.06; ‡*p* = 0.08; *p ≤ 0.05.

To determine if AhR-mediated regulation of *Il22* was conserved at the level of protein, purified CD4^+^ T-cells or whole splenocytes were cultured with or without TCDD for 48 h under Th17 polarizing conditions. IL-22 was measured in CD4^+^ T-cells and in culture supernatants. As shown in Figure 5A, a small population of CD4^+^ T-cells was found to express IL-22 in vehicle-treated cultures. Addition of TCDD increased the percentage of IL-22^+^ cells (*p* = 0.06) and the amount of IL-22 produced on a per cell basis (*p* = 0.08) as measured by the median fluorescence intensity (MFI). IL-22 was also detected in supernatants from cultures of purified CD4^+^ T-cells. TCDD-treated samples had an average of 28 pg/ml compared to <8 pg/ml (the reporting limit) in vehicle-treated cultures (Figure 5B). Likewise, when whole splenocytes were cultured, treatment with TCDD increased the frequency of IL-22^+^ CD4^+^ T-cells (*p* = 0.05) and the MFI (*p* < 0.001; Figure 5C). Significantly more IL-22 was found in supernatants from whole splenocytes consistent with a previous report (Alam et al., 2010). Treatment with TCDD significantly increased the amount of IL-22 present (*p* = < 0.001) from 65 pg/ml in vehicle-treated cultures to 374 pg/ml in TCDD-treated cultures (Figure 5D).

Other statistically significant changes in gene expression induced by TCDD in differentiating CD4^+^ T-cells are summarized in Table 4. Increased expression of *Ctla4* (2.3-fold), *Cd40lg* (2.2-fold), and *Il12rb2* (2.9-fold) was associated with TCDD treatment at 24 h under Th1 conditions. *Il12rb2* was also increased (7.1-fold) by TCDD under Th17 conditions at 48 h. Under Treg conditions, only *Stat4* expression was altered by TCDD and it was down-regulated (−2.7-fold), while no changes were seen under Tr1 conditions. Under Th0 conditions, TCDD significantly suppressed expression of *Tbet* (2.7-fold) and *Il17a* (2.1-fold).

**Table 4:** **Summary of changes in expression of immune-related genes induced by TCDD under different polarizing conditions**.

Polarizing condition	24 h TCDD/vehicle			48 h TCDD/vehicle		
	Gene	Fold change	*p*-value	Gene	Fold change	*p*-value
Th0	*Il17a*	−2.1	0.00	No significant changes		
	*Tbet*	−2.7	0.05			
Th1	*Il12rb2*	2.9	0.06	No significant changes		
	*Ctla4*	2.3	0.02			
	*Cd40lg*	2.2	0.02			
Treg	No significant changes			*Stat4*	−2.7	0.01
Tr1	No significant changes			No significant changes		
Th17	No significant changes			*Il12rb2*	7.1	0.03

*Data are presented as fold change in TCDD-treated cells relative to vehicle-treated controls at 24 or 48 h. None of the changes shown were observed in AhR^−/−^ CD4^+^ T-cells exposed to TCDD. Data represent 3–4 biological replicates*.

### Influence of AhR deficiency on gene expression in activated CD4^+^ T-cells

**Figure 6:**
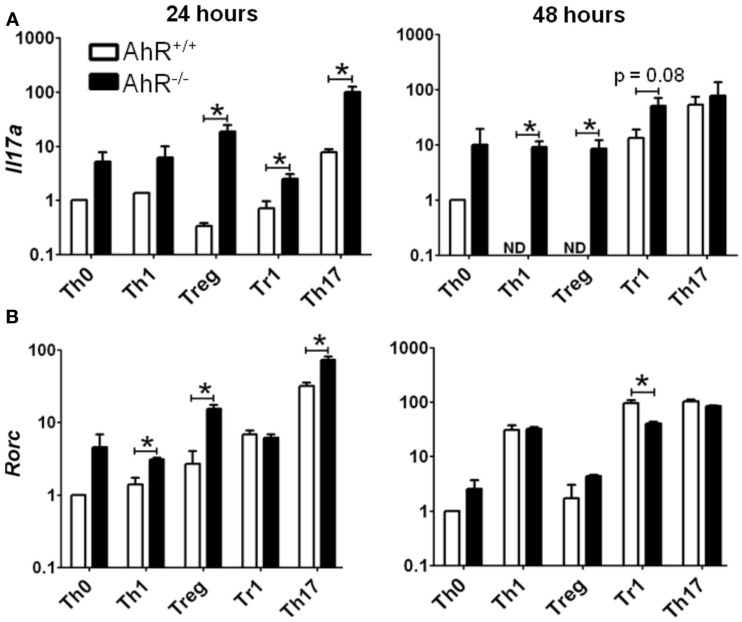
**Influence of AhR deficiency on expression of (A) *Il17a* or (B) *Rorc* in AhR^+/+^ or AhR^−/−^ CD4^+^ T-cells cultured under different polarizing conditions for 24 or 48 h**. Data are expressed as fold change relative to AhR^+/+^ T-cells cultured under vehicle-treated Th0 conditions. Error bars represent the mean ± SEM; *n* = 3 biological replicates, except for 24 or 48 h AhR^−/−^ Treg conditions, and 24 h Th17 conditions, where *n* = 2. *p ≤ 0.05. ND = not detected within 40 cycles.

Significant changes in gene expression were observed between vehicle-treated AhR^+/+^ and AhR^−/−^ CD4^+^ T-cells cultured under different polarizing conditions (Figure 6). The most significant effect of AhR deficiency was increased expression of *Il17a* at both 24 and 48 h under all culture conditions (Figure 6A). The greatest up-regulation of *Il17a* was noted under Treg conditions, with a 53-fold increase, whereas 16- and 4-fold changes were observed under Th17 and Tr1 conditions, respectively, at 24 h, with a similar trend for Th1 and Th0 conditions. At 48 h, *Il17a* expression was significantly up-regulated in AhR^−/−^ CD4^+^ T-cells under Th1, Treg, and Tr1 conditions.

Aryl hydrocarbon receptor deficiency also resulted in increased expression of *Rorc*, the master transcription factor for Th17 differentiation (Figure 6B). At 24 h, AhR^−/−^ T-cells expressed a sevenfold increase in *Rorc* under Treg conditions, and a twofold increase under Th1 and Th17 conditions. *Rorc* expression was also increased in AhR^−/−^ cells under Th0 conditions (*p* = 0.06), while expression was unaltered under Tr1 polarizing conditions. AhR regulation of *Rorc* was no longer seen at 48 h, with the exception of Tr1 conditions, where *Rorc* was down-regulated twofold in AhR^−/−^ CD4^+^ T-cells.

**Figure 7:**
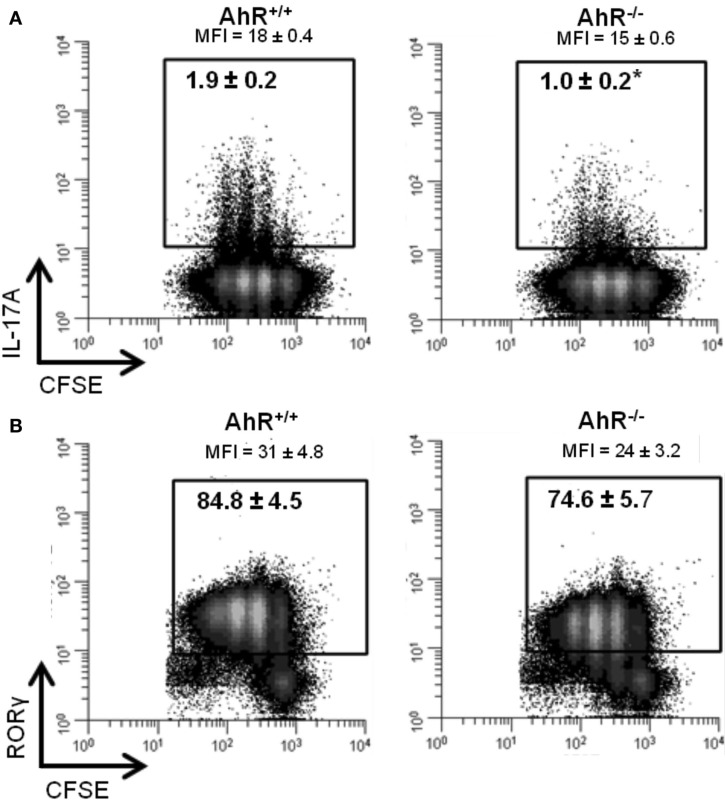
**Influence of AhR deficiency on (A) IL-17A or (B) RORγ protein in AhR^+/+^ or AhR^−/−^ CD4^+^ T-cells cultured under Th17 polarizing conditions for 48 h**. Data shown are gated on viable CD4^+^ T-cells. Percent IL-17A^+^ or RORγ^+^ T-cells are shown. MFI, median fluorescence intensity. Histograms shown are representative of 3−4 biological replicates. *p ≤ 0.05. Changes in RORγ expression were not statistically significant.

To determine if the increase in expression of *Il17a* and *Rorc* was observed at the protein level, IL-17A, and RORγ were measured by flow cytometry in cells cultured under Th17 conditions for 48 h. As shown in Figure 7A, 1.9% of the AhR^+/+^ CD4^+^ T-cells expressed IL-17A compared to 1.0% of the AhR^−/−^ CD4^+^ T-cells (*p* = 0.05). MFI values were also lower in AhR^−/−^ cells (*p* = 0.1). Similarly, a smaller percentage of AhR^−/−^ CD4^+^ T-cells stained positive for RORγ compared to AhR^+/+^ CD4^+^ T-cells, but the changes were not statistically significant (*p* = 0.2; Figure 7B). The basis for the lack of correlation between gene and protein expression is not known but may be related to the transient nature of the up-regulation of *Il17a* and *Rorc* expression seen in AhR-deficient cells. Under Th17 conditions, this effect was most pronounced at 24 h but gone by 48 h. As expected, there was no effect of AhR status on CD4^+^ T-cell proliferation, assessed by CFSE dilution.

**Figure A2:**
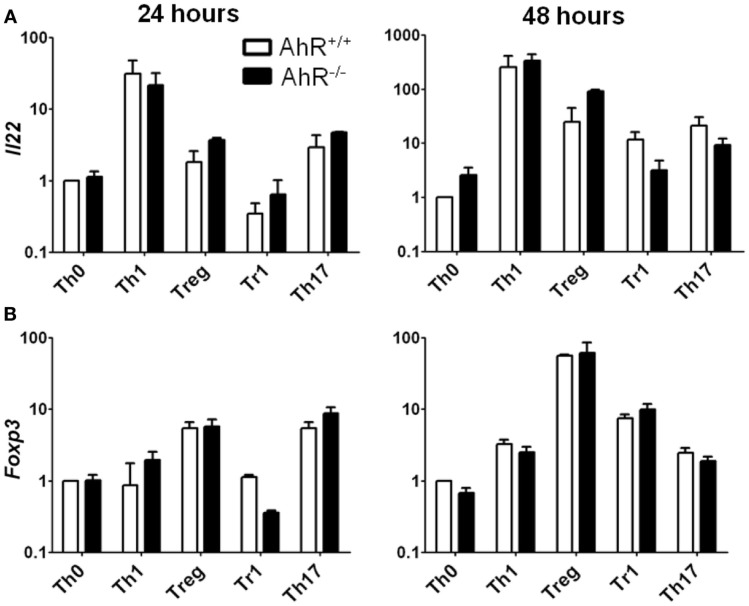
**Expression of *Il22* and *Foxp3* in AhR^+/+^ and AhR^−/−^ CD4^+^ T-cells cultured under different polarizing conditions**. Data are presented as fold change calculated relative to AhR^+/+^ T-cells cultured under vehicle-treated Th0 conditions. Expression of **(A)** *Il22* and **(B)** *Foxp3* in AhR^+/+^ and AhR^−/−^ cells at 24 and 48 h. Error bars represent the mean ± SEM; *n* = 3 biological replicates except for 24 and 48 h AhR^−/−^ Treg conditions, and 24 h Th17 polarizing conditions, where *n* = 2. *p ≤ 0.05.

Aryl hydrocarbon receptor deficiency had no significant effect on expression of *Il22* or *Foxp3* under any of the polarizing conditions tested (Figure A2 in Appendix). *Tgfb3* and *Gzmb* were up-regulated under Treg and Th0 conditions and *Il6* was up-regulated under Treg and Tr1 conditions in AhR*-*deficient cells, while other changes were unique to specific culture conditions (Table 5). In general, the results suggest that AhR deficiency in CD4^+^ T-cells favors increased expression of genes associated with inflammation, consistent with the pro-inflammatory phenotype of AhR-deficient mice.

**Table 5:** **Summary of changes in expression in immune-related genes related to AhR deficiency in CD4^+^ T-cells cultured under different polarizing conditions**.

Polarizing condition	24 h AhR^−/−^/AhR^+/+^			48 h AhR^−/−^/AhR^+/+^		
	Gene	Fold change	*p*-value	Gene	Fold change	*p*-value
Th0	*Nfatc2*	2.9	0.04	*Tgfb3*	6.0	0.01
	*Cd27*	−2.2	0.05	*Gzmb*	2.4	0.02
				*Cd69*	−2.1	0.02
Th1	*Ox40*	2.6	0.01	No significant changes		
	*Cd25*	2.3	0.05		
Treg	*Il2*	6.0	0.02	*Ifng*	3.9	0.04
				*Gzmb*	3.6	0.01
				*Tgfb3*	3.1	0.05
				*Il6*	3.0	0.05
				*Cd25*	2.1	0.05
				*Cd69*	−3.0	0.02
				*Stat4*	−5.8	0.03
Tr1	No significant changes			*Il6*	3.4	0.04
				*Tgfbr1*	2.1	0.01
				*Adora2b*	2.2	0.00
Th17	No significant changes			*Il2*	−5.0	0.01

*Data are presented as fold change in AhR^−/−^ CD4^+^ T-cells relative to AhR^+/+^ CD4^+^ T-cells at 24 or 48 h. Data represent 3–4 biological replicates, except for AhR^−/−^ cells at 24-h Treg and Th17 and 48-h Treg conditions where *n* = 2*.

## Discussion

The AhR is a ligand-activated transcription factor that has been shown to play a role in CD4^+^ T-cell differentiation, yet little is known about the changes in gene expression that occur in these cells following AhR activation. Previous studies have examined gene expression in donor CD4^+^ T-cells isolated from the spleen of TCDD-treated mice during a GVH response, however the identification of direct gene targets was confounded by the presence of contaminating host cells that also express AhR (Marshall et al., 2008). Identification of gene targets has become more important as recent reports indicate that there may be ligand-specific effects of AhR activation on CD4^+^ T-cell differentiation (Quintana et al., 2008; Veldhoen et al., 2008). Furthermore, the initial report of highly increased AhR expression in CD4^+^ T-cells cultured under Th17 conditions (Kimura et al., 2008; Veldhoen et al., 2008) led to an early misconception that AhR was functional only in Th17 cells in the mouse (Ho and Steinman, 2008; Veldhoen et al., 2008; Stockinger, 2009; Ramirez et al., 2010). However, more recently, AhR was shown to be significantly up-regulated in mouse and human CD4^+^ T-cells under Tr1 conditions as well (Apetoh et al., 2010; Gandhi et al., 2010). Given that TCDD suppresses Th1-, Th2-, and Th17-mediated responses *in vivo*, it implicates a role for AhR in the differentiation of many T-cell subsets. The present studies were designed to determine if genes involved in CD4^+^ T-cell differentiation are selectively regulated by AhR when the cells are activated in the presence of TCDD under a variety of Th-polarizing culture conditions.

The results of our studies show that *Ahr* is expressed in CD4^+^ T-cells under all conditions tested, including un-activated cells, while elevated levels of *Ahr* are seen under Treg, Tr1, and Th17 conditions. The highest level of *Ahr* expression was seen under Th17 conditions at 24 h and Tr1 conditions at 48 h. Functional AhR protein was also present under all culture conditions based on the ability of TCDD to induce the expression of known AhR-regulated genes (*Cyp1a1*, *Cyp1b1*, and *Ahrr*). The level of induced gene expression was generally proportional to the amount of *Ahr* present with the highest induction seen under Th17 conditions. Since many putative endogenous AhR ligands have anti-inflammatory functions, it suggests that up-regulation of AhR may be a protective response during highly inflammatory Th17 conditions, allowing low-affinity endogenous AhR ligands to down-regulate the response, consistent with the effects seen with TCDD. Examples of low-affinity ligands with immunosuppressive activity include lipoxin A_4_, bilirubin, and kynurenine (Sinal and Bend, 1997; Schaldach et al., 1999; Liu et al., 2008; Mezrich et al., 2010). Indeed, bilirubin and kynurenine have been shown to suppress EAE and induce CD4^+^Foxp3^+^ T-cells, respectively (Liu et al., 2008; Mezrich et al., 2010). Elevated levels of AhR in CD4^+^ T-cells undergoing Tr1 and Treg differentiation would also facilitate AhR signaling to promote the development of these anti-inflammatory cells.

TCDD did not appear to alter early polarization of activated CD4^+^ T-cells despite strong activation of AhR. Both vehicle- and TCDD-treated cells showed the expected increase in expression of *Tbet*, *Foxp3*, *Maf*, and *Rorc* under Th1, Treg, Tr1, and Th17 polarizing conditions, respectively. Likewise, the expression of other genes that are associated with specific polarizing conditions was unaffected by TCDD, including *Ifng*, *Cd39*, *Il10*, and *Il17a*. The lack of effect of TCDD on *Foxp3* expression was somewhat surprising since TCDD has been previously reported to directly regulate Foxp3 expression *in vitro* and increase the generation of Foxp3^+^ cells (Kimura et al., 2008; Quintana et al., 2008). However, the lack of effect of TCDD on *Foxp3* expression is consistent with the Foxp3^neg^ Treg phenotype induced by TCDD *in vivo* (Funatake et al., 2005; Marshall et al., 2008).

Only a few of the other genes in our 48-gene panel showed altered expression as a result of the presence of TCDD during CD4^+^ T-cell activation. *Ctla4* expression was increased by TCDD under Th1 conditions, a change that is consistent with the AhR-dependent increase in expression of CTLA-4 on donor CD4^+^ T-cells *in vivo* during the GVH response (Funatake et al., 2005). Likewise, increased expression of *Il12rb2* under both Th1 and Th17 conditions is consistent with the increased responsiveness of donor CD4^+^ T-cells from TCDD-treated mice to IL-12, resulting in enhanced STAT4 phosphorylation (Marshall et al., 2008). Under Th0 conditions, TCDD also suppressed the expression of *Tbet* and *Il17a*, providing a mechanism to promote Treg differentiation at the expense of effector Th1 or Th17 cells.

The most noteworthy effect of TCDD in CD4^+^ T-cells was the AhR-dependent increase in expression of *Il22* that was seen across all culture conditions except Tr1. AhR-mediated up-regulation of *Il22* was also observed at the protein level in CD4^+^ T-cells cultured under Th17 conditions. AhR signaling has been implicated in IL-22 production in previous studies (Veldhoen et al., 2008; Ramirez et al., 2010; Rutz et al., 2011). Additionally, Notch signaling was shown to create AhR ligands that enhance IL-22 production (Alam et al., 2010). Based on the fact that IL-22 can be produced in the absence of IL-17A or IFNγ, a unique Th22 subset of CD4^+^ T-cells has been proposed (Trifari et al., 2009). The putative transcription factor for this Th22 subset is the AhR (Trifari et al., 2009). IL-22 has complex functions and has been shown to produce both inflammatory (psoriasis, rheumatoid arthritis) and protective (irritable bowel disease) responses (reviewed in Zenewicz and Flavell, 2011). Enhanced production of IL-22 by CD4^+^ T-cells following AhR activation by TCDD may play a role in the immunoregulatory effects of TCDD.

Apart from its role in mediating effects of exogenous ligands such as TCDD, the consequences of AhR activation via endogenous ligands is also of interest. Although *in vivo* studies indicate that AhR^−/−^ mice generate normal adaptive immune responses to model antigens (Vorderstrasse et al., 2001) and that AhR^−/−^ donor CD4^+^ T-cells differentiate normally during an acute GVH response (Kerkvliet et al., 2002; Funatake et al., 2005), AhR-deficient mice have been reported to express a hypersensitive phenotype in experimental colitis, and following challenge with either LPS or cigarette smoke (Thatcher et al., 2007; Sekine et al., 2009; Furumatsu et al., 2011). Since several putative endogenous AhR ligands have been associated with the induction of immunosuppressive Tregs, the absence of AhR could impair Treg differentiation resulting in hyper-inflammation. Several pro-inflammatory genes were up-regulated in AhR-deficient cells at 48 h under Treg polarizing conditions including *Ifng*, *Gzmb*, *Tgfb3*, and *Il6* suggesting that the absence of AhR signaling in CD4^+^ T-cells alters Treg differentiation. Also consistent with a hypersensitive phenotype, CD4^+^ T-cells from AhR^−/−^ mice showed a transient increase in expression of genes associated with Th17 differentiation, *Rorc*, and *Il17a*, which was seen under Th1, Treg, Tr1, and Th17 conditions at 24 h. However, neither transcript nor protein levels were affected at 48 h when measured under Th17 conditions. These results suggest that the impairment of Th17 differentiation in the absence of AhR (Kimura et al., 2008; Veldhoen et al., 2009) results from post-transcriptional events. Similarly, whereas Kimura et al. (2008) reported that fewer Foxp3^+^ cells were generated when AhR^−/−^ cells were cultured under Treg conditions, AhR deficiency did not influence *Foxp3* expression under any of the conditions tested in our studies.

Given the emerging role of AhR in IL-22 production and the significant up-regulation of *Il22* by TCDD, we were surprised to find that AhR deficiency did not impair expression of the *Il22* gene. This finding was in direct contrast to the results of Veldhoen et al. (2008) who first reported that IL-22 expression was absent in AhR-deficient cells (Veldhoen et al., 2008). One possible explanation for these divergent findings may derive from the type of media used for T-cell culture. All of our studies utilized RPMI 1640 whereas Veldhoen et al. (2008) may have used a specific medium (IMDM) that contains high levels of aromatic amino acids including tryptophan, a source of several AhR ligands (Veldhoen et al., 2009). If IMDM was used, the expression of *Il22* may have been up-regulated in WT cells due to the presence of endogenous AhR ligands, while AhR-deficient cells would be unable to respond. We saw this effect when we compared IL-22 expression in Th17-polarized cultures of AhR^+/+^ and AhR^−/−^ CD4^+^ T-cells treated with TCDD. Expression of *Il22* was more than 50-fold higher in AhR^+/+^ cells at 48 h (unpublished observations). Our data suggest that AhR is not needed for constitutive IL-22 expression.

In summary, strong activation of AhR by TCDD in differentiating CD4^+^ T-cells failed to influence the expression of numerous genes associated with T-cell activation and differentiation. Since many of these genes have been shown to be altered by TCDD in differentiating CD4^+^ T-cells *in vivo*, the results cast doubt on the ability of *in vitro* conditions to recapitulate *in vivo* events. This could be due to lack of accessory cell signals not mimicked by antibodies to CD3 and CD28, or to the presence of high levels of polarizing cytokines that might dominate the signaling pathways. Other than known AhR-regulated genes (*Cyp1a1*, *Cyp1b1*, *Ahrr*), the only immune-related gene that was clearly regulated by AhR in CD4^+^ T-cells was *Il22*, a gene that has been recently associated with the AhR pathway. The possible role that IL-22 plays in the immunoregulatory effects of TCDD awaits future studies.

## Conflict of Interest Statement

The authors declare that the research was conducted in the absence of any commercial or financial relationships that could be construed as a potential conflict of interest.
